# RNA fluorescence in situ hybridization (FISH) as a method to visualize bacterial colonization in the *C. elegans* gut

**DOI:** 10.17912/micropub.biology.001044

**Published:** 2024-02-27

**Authors:** Kayla M Poirier, Robert J Luallen, Dalaena E Rivera

**Affiliations:** 1 Department of Biology, San Diego State University, San Diego, California, United States

## Abstract

*Caenorhabditis elegans* is an excellent model to study host-microbe interactions as it is easy to visualize bacterial presence in their intestine. However, previous studies have shown that utilizing transgenic, fluorescent protein-expressing bacteria is not a reliable method to distinguish living bacteria from dead bacteria in the lumen of *C. elegans* . In this study, we compared methods for visualizing bacterial presence within the *C. elegans* intestine and found that RNA f luorescent i n s itu h ybridization (RNA FISH) could distinguish the difference between intact and dead bacteria. Thus, we propose RNA FISH as the preferred method to visualize live bacterial presence in the intestines of *C. elegans* prior to fixation.

**Figure 1. Images and quantification of bacteria in day 2 adult C. elegans N2 measured by bacterial fluorescence and FISH:**
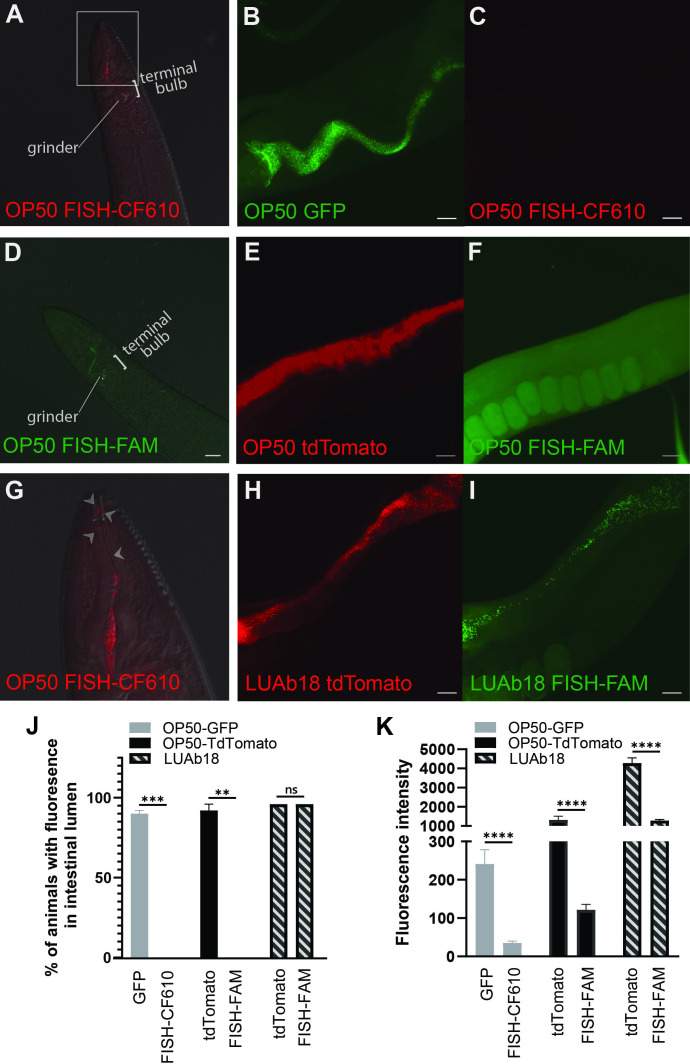
**(A, D)** N2 fed *E. coli* OP50 and FISH stained with species specific probes containing a red or green fluorophore, respectively, showing the presence of OP50 in the pharynx and grinder of the terminal bulb, but not in the intestine. **(B-C)** N2 fed *E. coli* OP50-GFP and FISH stained with species specific probes containing a red fluorophore, CF610. **(E-F)** N2 fed *E. coli* OP50 tdTomato and FISH stained with species specific probes containing a green fluorophore, FAM. **(H-I)** N2 fed a known colonizing bacteria LUAb3 tagged with tdTomato (LUAb18) and FISH stained with species specific probes containing a green fluorophore, FAM. Scale bars are 20 μm. Note: B and C, E and F, and G and H are the same animal. **(G)** Inset for white boxed region in **A** . Arrows indicate individual bacilli as detected by the *E. coli* OP50 CF610 RNA FISH probe. **(J)** Percentage of worms with fluorescent signal detected in the intestinal lumen. Results are from n=25 over 2 independent experiments, where p<0.01 (**), p<0.001 (***), and ns is non-significant by unpaired two-tailed t-test. **(K)** Quantification of the fluorescence intensity between fluorescent protein tagged and FISH stained N2 fed OP50 GFP, OP50 tdTomato, and LUAb18, respectively. Results are from n=25 examined over 2 independent experiments, p<0.0001 (****) by unpaired two-tailed t-test.

## Description

*Caenorhabditis elegans* has emerged as an excellent model to study host-microbe interactions due to their genetic tractability and transparent bodies that allow for easy visualization of microbial infection and colonization. Additionally, the *C. elegans* intestinal cells have morphological and functional similarities to those of other animals, including vertebrates (Brenner, 1974; Pukkila-Worley and Ausubel, 2012; Balla and Troemel, 2013; Zhang and Hou, 2013) . The gut microbiome of *C. elegans* is naturally comprised of microbes that form a niche in the intestinal lumen *.* One common method to visualize bacterial presence in the intestine is through feeding *C. elegans* fluorescently tagged bacteria and observing fluorescence in the lumen. However, studies have shown that fluorescence alone cannot distinguish dead from live, intact bacteria, with GFP-expressing *E. coli* OP50-1 showing fluorescence in the gut lumen despite TEM showing no intact bacteria (Hsiao et al., 2013) . This discrepancy can confound experiments that study microbiome colonization of the *C. elegans* gut when they utilize fluorescently tagged transgenic bacteria, potentially leading to incorrect conclusions on the capacity of bacteria to form a niche and replicate in the gut lumen.

Our lab studies microbiome bacterial colonization of the *C. elegans* gut utilizing natural bacteria that can adhere to intestinal cells in the lumen (Morgan et al., 2021) . A key aspect of bacterial colonization is the ability to remain alive and intact in the gut lumen. To better visualize and quantify these gut microbes, we commonly use RNA f luorescence i n s itu h ybridization (FISH). We have previously described this technique in Rivera et al., 2022. RNA FISH utilizes fluorescent DNA probes that are complementary to the highly expressed 16S ribosomal RNA sequence allowing for visualization of bacteria of interest in *C. elegans* (Rivera et al., 2022) . Because these probes bind to 16S rRNA, they are a better indicator of the presence of intact bacteria since rRNA from dead bacteria would quickly degrade in the intestinal lumen. Thus, the fluorescence seen in bacteria via FISH would likely represent a method to quantify live bacterial presence in the *C. elegans* lumen, before fixation during the FISH procedure. We suggest RNA FISH is a better tool to visualize the presence of live bacteria, rather than relying on GFP- or tdTomato- tagged bacteria alone.

We compared the detection of bacteria in the *C. elegans* gut lumen using either transgenic fluorescent bacteria or RNA FISH. We first fed *C. elegans* N2 with *E. coli* OP50-GFP or *E. coli* OP50 -tdTomato for 96 hours. We then fixed the animals and performed RNA FISH using species-specific FISH probes with a contrasting-colored fluorophore. For example, OP50-GFP samples were stained using an *E. coli-* specific FISH probe containing a red fluorophore, CF610. OP50 should not consistently be present in the lumen of young, healthy *C. elegans* adults (Darby, 2005) . By contrast, we have isolated a natural microbiome bacterium, *Lelliottia jeotgali* (LUAb3), that adheres and colonizes the *C. elegans* intestines. We created a LUAb3 strain with transposon mediated insertion of tdTomato (LUAb18) to use as a positive control to visualize live bacteria in the intestine. Though the animals are fixed during the RNA FISH process, they represent the state of the intestine before fixation.

Our results showed that animals fed with OP50-GFP had no detectable red fluorescent FISH signal (CF610) in the lumen ( Fig. 1C ), consistent with the expectation that OP50 is not present in the *C. elegans* lumen. As an internal control, we found that *E. coli* can be detected in the pharynx of some fixed worms via FISH, but the host grinder within the terminal bulb efficiently crushes the bacteria resulting in no FISH signal downstream in the intestine ( Fig. 1A and 1G). By contrast, there was detectable GFP in the intestine of the same fixed animal ( Fig. 1B ). This green fluorescent signal could be misleading and is not indicative of bacterial presence because OP50 does not naturally colonize the intestinal tract of *C. elegans* (Darby, 2005) . When we quantified this detection, we found ~90% of animals had transgenic GFP signal while 0% of animals had FISH (CF610) signal in the intestinal lumen (Fig 1J, left).

When we flipped the fluorophore colors, we saw similar results. Here, we fed OP50 -tdTomato to animals and saw red signal detected in the intestinal lumen of fixed animals ( Fig. 1E ). By contrast, RNA FISH using a green fluorescent probe (FAM) showed no green fluorescence in the lumen ( Fig. 1F ). As an internal control, we found that *E. coli* can be detected in the pharynx of some fixed worms via FISH, prior to destruction in the grinder ( Fig. 1D ). Similar to before, we found ~92% of animals had detectable transgenic tdTomato signal in the lumen, while 0% of animals had green FISH (FAM) signal (Fig 1J, middle).

Finally, we tested a natural *C. elegans* microbiome bacterium, *L. jeotgali* , that adheres to and colonizes the intestinal lumen. A tdTomato-expressing *L. jeotgali* strain, LUAb18, was fed to animals and fixed for FISH using a specific, FAM-labeled probe. We observed that animals had both red and green fluorescent signal in the lumen ( Fig. 1H -1I). From quantification, we found that FISH fluorescence and tagged fluorescence were each detected in ~95% of animals, suggesting that LUAb18 was live and intact in the lumen prior to fixation ( Fig. 1J, right ).

When we quantified these experiments using average fluorescence intensity, we found that the results largely matched our observations, with fluorescent-tagged *E. coli* showing significantly higher fluorescence than FISH-stained bacteria, which was only slightly higher than the background ( Fig. 1K ). This suggests that transgenic *E. coli* have residual fluorescence in the lumen despite the absence of intact bacteria. By contrast, LUAb18 bacteria had high fluorescence intensity regardless of the method of detection (FISH vs transgenics). However, there was still a significant difference between the two methods in LUAb18, likely due to residual tdTomato protein which has a reported half-life of greater than 72 hours (Muzumdar et al., 2007) , indicating the presence of both live and dead bacteria in the intestines of LUAb18 fed animals before fixation.

The stability and persistence of GFP prevents accurate detection of live bacteria, thus emphasizing that using GFP- and RFP-tagged bacteria may not be a reliable method to visualize bacterial presence. In vivo, GFP has a reported half-life longer than 24 hours (Andersen et al., 1998) , resulting in residual GFP signal in the intestine though the bacteria are dead. Alternatively, unstable GFP variants with shorter half-lives may better reflect live bacterial expression within the host (Tombolini et al., 1997; Andersen et al., 1998; Li et al., 1998; Leveau and Lindow, 2001) . RNA FISH is sensitive enough to detect a single bacterium as seen in Fig. 1G . These RNA FISH probes rely on targeting the small ribosomal subunit of bacteria, which comprises nearly 80-90% of the total RNA in the cell (O'Neil et al., 2013), resulting in robust staining in intact bacteria. Because ribosomal RNA is generally stable and protected within intact bacteria (Neidhardt, 1964; Deutscher, 2009; Blazewicz et al., 2013; Sulthana et al., 2016) , RNA FISH probes can readily hybridize to their target sequences. The absence of viable 16S rRNA in dead bacteria may be due to the fast degradation of RNA from environmental RNases released from bacteria killed in the host grinder (Deutscher, 2009; Deutscher, 2015; Sulthana et al., 2016; Bechhofer and Deutscher, 2019) . Overall, we demonstrate that solely using GFP, tdTomato, and/or other stably expressed fluorophores in bacteria is not sufficient to indicate the presence of live bacteria in *C. elegans* . We suggest RNA FISH as a more reliable method to accurately visualize and detect intact bacteria in the *C. elegans* intestine.

## Methods

An overnight LB broth culture of each bacterial strain of interest ( OP50 -tdTomato, OP50-GFP , or LUAb18) was seeded onto a 10 cm plate of NGM agar containing 50 μg/mL of carbenicillin. Wild type *C. elegans* N2 were maintained on Nematode Growth Media (NGM) plates seeded with *Escherichia coli* OP50 incubated at 20° C. Once N2 reached gravid state, the nematodes were bleached for 1-2 minutes with sodium hypochlorite and 5M NaOH to extract the eggs (Stiernagle, 2006) . The sodium hypochlorite and NaOH were removed through a series of M9 washes and the eggs were left to hatch and develop into L1s overnight in M9. The bleached and synchronized L1s were plated and fed with LUAb18, OP50-GFP , or OP50 -RFP.

After 96 hours, the animals were fixed with paraformaldehyde for 30 min and FISH stained as described previously (Rivera et al., 2022) . The FISH probes used were designed to the 16S rRNA of bacteria and conjugated to either 5-Carboxyfluorescein (FAM) or CAL Fluor Red 610 (CF610). LUAb18 was stained with the probe b003_16S_A targeting the 16S rRNA sequence CTCTCTGTGCTACCGCTCG. OP50 , OP50-GFP and OP50 -RFP were stained with the probe OP50_16S_A with the sequence CAGCGAAGCAGCAAGCTGC. Images were taken using a fluorescent Eclipse Ni microscope (Nikon) at 40x magnification and the exposure time was consistent for all images. Exposure times for the GFP channel was 800 ms and RFP channel was 2 s for all images. To quantify the percentage of worms with fluorescence in the intestines, we observed the fixed images and counted the number of worms colonized, which we defined as the presence of any fluorescent signal in the intestinal lumen. To perform statistical analysis on this data, we introduced variance by adding 0.001 to values with no variance. Fluorescence intensity was quantified using FIJI (Version: 2.14.0/1.54f) as previously conducted (Schindelin et al., 2012; Rexxoagli et al., 2019) . Statistical analyses were performed using Graphpad Prism (version 10.1.0 (316)).

## Reagents

**Table:** 

strain	Species type	Host Strain	genotype	Available from
N2	*Caenorhabditis elegans* wild type		Wild type	CGC funded by NIH Office of Research Infrastructure Programs (P40 OD010440)
OP50-1	*Escherichia coli* wild type		Wild type	CGC funded by NIH Office of Research Infrastructure Programs (P40 OD010440)
OP50 -tdTomato	*Escherichia coli*		Wild type + A22 tdTomato-expressing OP50 cloned into pGEX-5x-3 vector TAC promoter	CGC funded by NIH Office of Research Infrastructure Programs (P40 OD010440)
OP50-GFP	*Escherichia coli*		Wild type + GFP plasmid (pFPV25.1 rpsM promoter)	CGC funded by NIH Office of Research Infrastructure Programs (P40 OD010440)
LUAb3	*Lelliottia jeotgali*	*C. elegans* (LUA21)	Wild strain	Isolated from a rotting giant leopard plant stem ( *Ligularia tussilaginea* ) on SDSU campus, San Diego CA on March 18, 2019.
